# Turning tables: food availability shapes dynamic aggressive behaviour among asynchronously hatching siblings in red kites *Milvus milvus*

**DOI:** 10.1098/rsos.230328

**Published:** 2023-07-19

**Authors:** Benedetta Catitti, Urs G. Kormann, Valentijn S. van Bergen, Martin U. Grüebler

**Affiliations:** ^1^ Swiss Ornithological Institute, Seerose 1, 6204 Sempach, Switzerland; ^2^ Department of Evolutionary Biology and Environmental Studies, University of Zurich, Zurich, Switzerland

**Keywords:** competition, aggression strategies, dominance, network analysis, sibling competition, brood reduction

## Abstract

Aggression represents the backbone of dominance acquisition in several animal societies, where the decision to interact is dictated by its relative cost. Among siblings, such costs are weighted in the light of inclusive fitness, but how this translates to aggression patterns in response to changing external and internal conditions remains unclear. Using a null-model-based approach, we investigate how day-to-day changes in food provisioning affect aggression networks and food allocation in growing red kite (*Milvus milvus*) nestlings, whose dominance rank is largely dictated by age. We show that older siblings, irrespective of age, change from targeting only close-aged peers (close-competitor pattern) when food provisioning is low, to uniformly attacking all other peers (downward heuristic pattern) as food conditions improve. While food allocation was generally skewed towards the older siblings, the youngest sibling in the nest increased its probability of accessing food as more was provisioned and as downward heuristic patterns became more prominent, suggesting that different aggression patterns allow for catch-up growth after periods of low food. Our results indicate that dynamic aggression patterns within the nest modulate environmental effects on juvenile development by influencing the process of dominance acquisition and potentially impacting the fledging body condition, with far-reaching fitness consequences.

## 1. Introduction

Competition is a fundamental process regulating animal populations according to the available resources [1]. Within families, competition among growing siblings lies at the interface between the environment and parental care and regulates resource allocation in a way that maximizes their inclusive fitness [2,3]. A widespread but costly form of competition is physical aggression, which aims at establishing dominance over resources [4–7], and routinely leads to the elimination of the weakest sibling in some, but not all, bird species [8–10]. Acquiring a dominant position early on can have both short-term benefits, e.g. when fighting over resources [11,12] and long-term benefits, by shaping dominant personalities that may outperform the subordinate ones also later in life [13–15]. Although much theoretical and empirical work has been done to understand obligate and facultative brood reduction, the detailed mechanism through which sibling aggression shapes differential food intake in variable environments remains poorly understood. The mechanism is at the core of how variation in food provisioning translates into differential fledging conditions and dominant behaviour, and thus into within-family differences in individual quality at fledging [16].

In bird species living in environments characterized by variable food availability, clutch sizes are often larger than what parents eventually raise until fledging and asynchrony is favoured as an evolutionary strategy to create size-based hierarchies [17]. In agreement with game-theoretic models, the establishment of hierarchies in the early developmental stages helps to minimize aggressive interactions and hence energy wastage when a conflict arises [18–22]. However, even within the boundaries of asynchrony-dictated interactions, juveniles vary in the frequency and direction of their attacks [16]. In social groups, the decision to attack is influenced by the contestants' energy budgets [23]. It has been suggested that variable environmental conditions affect the occurrence of three alternative aggression patterns [24]: 1. aggressions from higher-rank individuals towards all lower-rank ones (downward heuristic), 2. aggressions among similar-rank individuals (close competitors) or 3. aggressions towards the lowest-rank individual (bullying). According to this framework, the environmental conditions that nestlings experience during development can affect the costs associated with aggression. Environmental conditions often change during the nestling period, and we expect temporal variation in aggression patterns as the nestlings grow. However, the dynamics of aggression patterns within broods due to environmental variability have been poorly investigated.

The effect of environmental conditions on aggression is expected to be modulated by individual characteristics. On one side, the competitive abilities of nestlings generally change over the nestling period, resulting in age-specific decisions to engage in aggressive interactions. On the other side, aggression patterns may also change due to the establishment of dominance–subordinance roles through learning processes involving winner–loser effects and the outcome of previous dyadic interaction [20,25–27]. This process usually translates into an overall decrease in the frequency of aggression and helps to minimize the energy loss associated with aggressive interactions while maximizing the inclusive fitness of each family member [28]. While recent theoretical work characterized how time-dependent cost-benefit trade-offs shape the emergence of hierarchies in animal societies [26,29], it remains unclear whether such mechanisms translate to aggression pattern dynamics in asynchronously hatching species.

In species where differences in nestling size strongly affect food competition, the dominance status is expected to feed back into growth, leading to progressively larger size differences [29]. Such behaviour-state feedback loops can have profound effects on the emergence of individuality in animals [30,31]. Environmental variation in food availability may trigger behaviours that either intensify or mitigate the effect of these loops. For instance, if food is scarce, senior siblings may adopt a ruthless attitude towards junior peers (bullying), which can even represent a source of food for the eldest (through cannibalism, ice-box hypothesis, [32]). Conversely, junior siblings may reach a tipping point where the benefits of challenging the dominant individual for a resource outweigh the costs [28], but this threshold may depend on their stage of development, which is closely linked to their survival probability (reproductive value; RVe, [28]). Therefore, fluctuations in environmental conditions are expected to have time-dependent effects on aggression patterns, and the timing of these changes may influence their impact. As a result, adopting a dynamic approach is crucial to understand the role of the environment in linking aggression in the nest to individual conditions.

The recent ascent of social network approaches in the field of behavioural ecology [33] has opened new possibilities to explore environmental effects on dyadic interactions. A network approach not only allows us to detect social structure and subtle changes to it but also to test whether an observed pattern deviates from what is expected in the absence of the pattern-generating process by implementing customized null models [34,35]. For example, recent applications of such network null models revealed cost-dictated interaction strategies of guinea fowls [23], but the avenues of this versatile methodology are numerous and remain little explored. Network approaches are well suited to study aggression patterns between nestlings, as they grow in a naturally enclosed space and thus the chance of each individual to interact with the others is fairly equal. Thus, they represent a promising tool to identify strategies at the brood level under changing environmental conditions in light of inclusive fitness.

In this study, we (i) investigate how red kite (*Milvus milvus*) nestlings alter the allocation of aggressions across hatching ranks along a natural food gradient during the nestling period, by examining aggression intensity and directionality and (ii) explore whether the variation in aggressive interactions translates into patterns of food partitioning among nestlings. Red kites are a facultative siblicidal species and exhibit marked hatching asynchrony, with the largest hatching interval occurring between the last two nestlings. Nestlings stay in the nest for 50 to 60 days, during which they are entirely dependent on their parents for food provisioning. For the first 20 to 30 days, parents (mostly the female) feed directly each nestling with small bouts until they develop the ability to autonomously tear apart entire prey items [36]. During these direct feeding events, aggression may strongly influence to which sibling the feeding bout is allocated [12,37]. Due to their size difference, marginal (last-hatched) nestlings have a competitive disadvantage which is usually protracted through development, as suggested by two previous studies showing that marginal nestlings incurred in high-stress levels [38] and low survival probability [39], but that these effects were dampened when supplementary food was provided to the brood. However, increased stress can be associated with contrasting behavioural responses [40], and thus contributes to the establishment of distinct dominance–subordinance personalities early in life. By investigating the aggression patterns and food distribution under changing food conditions during the nestling period, we aim to enhance our understanding of how environmental conditions translate into within-brood differences in quality during development and later in life.

## 2. Methods

### 2.1. Study species and area

Our study area extends for ca. 400 km^2^ across the Swiss cantons of Fribourg and Bern (N: 46°47′ 60″ E: 7°15′ 00″). Between the end of March and end of April, red kites lay one to three, rarely up to four eggs, which hatch after ca. 32 days. In broods of three, eggs are laid on average every 72 h, but the hatching interval can greatly vary: the first two nestlings usually hatch synchronously or at a short time interval, while between the second and the third individual a large hatching interval of up to 96 h can occur [41]. Red kites are opportunistic scavengers, either feeding on small vertebrates (largely rodents) or feeding on carcasses or various sources of anthropogenic food [42,43]. Two factors contribute to the large spatio-temporal variation that may occur in the red kite feeding behaviour. First, rodent availability is linked to agricultural activities, with mowing or ploughing events greatly facilitating the accessibility of prey for the red kites [36]. Second, anthropogenic food resources are not evenly distributed in time and space [42].

### 2.2. Video recording and analysis

To monitor the nestlings’ aggressive behaviour after hatching, we mounted 30 video surveillance systems in 2019 and 2020 through a stratified sampling design along the latitudinal gradient. Cameras were mounted before the beginning of egg laying either in known nests, or on nests where we observed nest-building activity. Across both years, a total of 18 successful broods (i.e. at least one chick fledged) were surveyed. Each video system was composed of (i) a closed-circuit-television camera (700TVL HD Bullet SONY Effio-E), (ii) a digital video recorder (DVR, Marbil Enterprise Inc.) connected to (iii) a 12-volt deep-cycle block battery (Lithium High Power Battery LiFePO4 (12,8V/20Ah), Nothnagel Marine Elektromechanik). The camera was screwed to a branch at an average distance of 1 metre from the nest (±45 cm) and connected to the DVR system on the ground. The DVR was set to record continuously between 8 AM and 9 PM (Central European Time; UTC + 1). Because the recording system was located at the bottom of the tree, the disturbance during the breeding period was minimal. Nestlings were individually marked with non-toxic fabric paint (three-dimensional fabric paint; Arteza) at the first climb (age of the oldest nestling = 8 days) to facilitate nestling identification during video analysis. Red-shaded colours were not used to avoid the impression of wounds which may have influenced aggressive behaviour. Before the first climb, it was possible to tell the nestlings apart by observation based on size differences. When that was not the case (*N* = 2 broods, always between the first two nestlings), interactions between the oldest siblings were given a separate code and we then randomly allocated either senior (S) or middle (M) as attacker and receiver.

### 2.3. Recording of aggressive interactions, food allocation and biomass delivered

For each nest, we selected four 4-day intervals based on the age of the oldest nestling (hatching day = 0): age 4–7 d, 11–14 d, 18–21 d, 25–28 d. Intervals between 8 am and 2 pm were manually analysed by the same observer each year (*N* total observers = 2) by recording the occurrence of specific behavioural events of nestlings and adults per second. This time interval was selected based on a preliminary analysis of a subset of broods (*N* = 5) indicating that most feeding and aggressive events occurred between 8 am and 2 pm (>90%, see the electronic supplementary material, figure S1). Specifically, we recorded the presence of the adults on the nest, prey deliveries, feeding and pecking events. Pecks were defined as physical attacks where one individual strikes the opponent, usually at the head or neck. During pecking events, both the active individual and the recipient of each peck were recorded. At each feeding event, we recorded the number of feeding bouts received by each individual, but due to time constraints, it was only recorded until the age of 21 d of the oldest sibling. To estimate the amount of biomass delivered per day, we grouped all the identified prey deliveries into six categories (amphibians, anthropogenic food, birds, invertebrates, mammals and unclassified). When possible, we recorded the proportion of prey that was delivered to the nest (whether it was whole, half, or one-quarter). We estimated the weight of each prey category in two ways. Firstly, we used a mass estimate that the observer provided based on visual inspection of the video frames. To reduce observer bias, both observers were provided with a list of standard prey items and the corresponding weights, together with raw data of measured prey in the field before the beginning of the video analysis. If no such weight data were available, we used the data from prey weighted during the nest controls between 2015 and 2021 to extrapolate an average weight per prey category (amphibians *N* = 11, anthropogenic food *N* = 95, birds *N* = 32, mammals *N* = 323), except for invertebrates which are promptly consumed upon their delivery, for which we estimated the weight from the literature [44].

### 2.4. Statistical analyses

#### 2.4.1. Tendency to peck

All analyses were performed in R (v. 4.05). To identify aggression patterns and to investigate whether they changed with the degree of asynchrony and food provisioning levels, we adapted a method developed by Dehnen *et al*. [23], which builds upon the work from Hobson *et al*. [27] where observed interaction frequencies are compared to frequencies expected under a permutation-based null model. To investigate the change of aggression patterns as nestlings grow, we split the dataset into a first and a second post-hatching period, starting from the hatching of the last nestling until the age of 10 d, and from the age of 10 until 20 d, after which period almost no aggression was recorded in the nests. The first period represents the most vulnerable time for the nestlings, whereas in the second period, the probability to survive is considerably higher [39]. We only selected pecking events in brood sizes of three (maximum brood size) to be able to randomize the attack recipients. In their study, Dehnen *et al*. examined whether individuals of varying ranks within a social group strategically displayed interactions with different costs. To avoid circularity, the researchers used a data-splitting approach to estimate rank differences independently of interaction rates. Here, we focused on whether high-cost interactions (pecking) are displayed strategically among individuals of different ages, ultimately shaping food distribution patterns and dominance over food resources. Hence, we defined the rank *a priori* as age differences and avoided circularity.

We calculated age differences for each pairwise observation, with negative values occurring when the aggressor was older than the recipient and positive ones when the actor was younger than the recipient. We allocated 80% of the data to estimate the relationship between pecking propensity and pairwise age difference. To quantify the uncertainty of this relationship, we bootstrapped this random split 100 times. A total of 100 permutations were considered sufficient as performing more permutations did not produce any change in the direction of the effects. For each of these 100 permutations, we calculated observed daily directed interaction frequencies. Next, we performed permutation tests on those daily interactions by randomly allocating (10 000 times) the interaction recipients from the pool of possible recipients—either the actual recipient or the other sibling. We then calculated the difference between observed and random interaction frequencies (i.e. the deviation from random interactions), thus generating a matrix describing the daily tendency to interact in each nest. To investigate whether variation in short-term food provisioning drives interaction patterns, we used a Bayesian regression mixed model using the ‘bamlss’ package [45] to model the deviation from random interactions as the response (Gaussian response distribution) and age differences in interaction with daily food provisioning (g) and phase (first/second) as a tensor product interaction term. We added brood ID as a random intercept to account for brood-specific aggression patterns. We fitted two separate models for positive and negative age, because individuals that hatched one day earlier or later than the other may differ substantially in their behaviour, and fitting a smooth term through the whole age difference spectrum can underestimate such differences. We extracted the predicted values from each bootstrap sample for every age difference value at the minimum (poor food conditions), median (average food conditions) and maximum (favourable food conditions) values of biomass. We then used this dataset of predicted values to derive the mean and 95% confidence intervals. Aggression between two siblings can escalate to intense combat due to the impossibility of the nestlings to physically leave the fighting arena. Escalation of conflicts may influence the decision to attack one sibling instead of the other and hence generate spurious results about the drivers of aggression patterns. To account for this, we applied the same method to a subset of the data where we selected only the first peck of each dyadic ‘battle’, but this yielded qualitatively the same results, with the exception of the aggression patterns of the younger siblings in high food conditions (see the electronic supplementary material, figure S2).

#### 2.4.2. Food allocation

To investigate the proportional changes in food allocation among siblings in relation to food provisioning, we fitted a Bayesian mixed, additive, multinomial logit regression model implemented in the ‘bamlss’ package. We differentiated between three nestling categories: senior (S), middle (M) and junior (J), indicating which of the three nestlings received the feeding bout. We separately examined how the probability of receiving a feeding bout changed in relation to the total biomass received each day and the daily dominant aggression pattern expressed in the nest. The dominant aggression pattern was calculated as the ratio between the attacks received by the middle and junior nestling each day. This peck ratio was log-transformed prior to modelling to achieve normality and ease interpretation. Because the senior nestling is usually the most aggressive, and the largest variation in age difference occurs between the junior nestling and two older siblings, a positive log ratio between the pecks received by M and J can be interpreted as a proxy for close competitor patterns. By contrast, a negative log ratio suggests a downward trend in aggressions along the age gradient (downward heuristic patterns). Similarly, when the senior nestling is the primary contributor of aggression, a log ratio close to zero may also imply analogous downward heuristic patterns. Total biomass and log-peck ratio were included as smoothers (*k* = 4 and *k* = 3, respectively), after comparing the Watanabe–Akaike information criterion (WAIC) with models with either lower or higher number of knots (Delta WAIC > 2). We further included the age of the oldest nestling also as a smoother to control for changes in food provisioning with age, and brood ID as a random intercept. Modelling age and biomass with smoothed terms allowed us to directly account for the temporal dependency of subsequent feeding bouts (serial autocorrelation) and to model potential nonlinear effects in a non-parametric framework.

## 3. Results

For the first and second post-hatching periods, we recorded a total of 5334 pecking events from 11 and 1055 pecking events from 7 broods of three nestlings, respectively. A total of 5999 feeding bouts were observed from 10 broods in the first period while for the second period, the feeding bouts recorded were 2622 from 3 broods.

### 3.1. Tendency to peck

**Figure 1.**
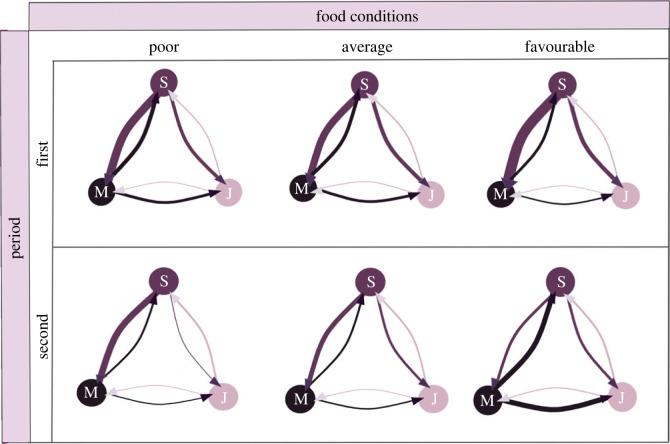
Aggression networks as a function of food conditions and post-hatching period in red kite broods of three. Nodes represent nestlings S (senior), M (middle) and J (junior). Edge widths represent the average number of pecks occurring in each dyad, which are squared-transformed to improve readability. The first phase (between age 1 and 10 of nestling J; panel above) has a higher average number of pecks per day than the second phase (between age 11 and 20 of nestling J; panel below), and the highest aggression is recorded under favourable food conditions in both periods. Under poor and average food conditions, the intensity of dyadic attacks is similar, while under favourable conditions, S increases its attacks in the first phase, while M becomes the main actor of attacks in the second one.

**Figure 2.**
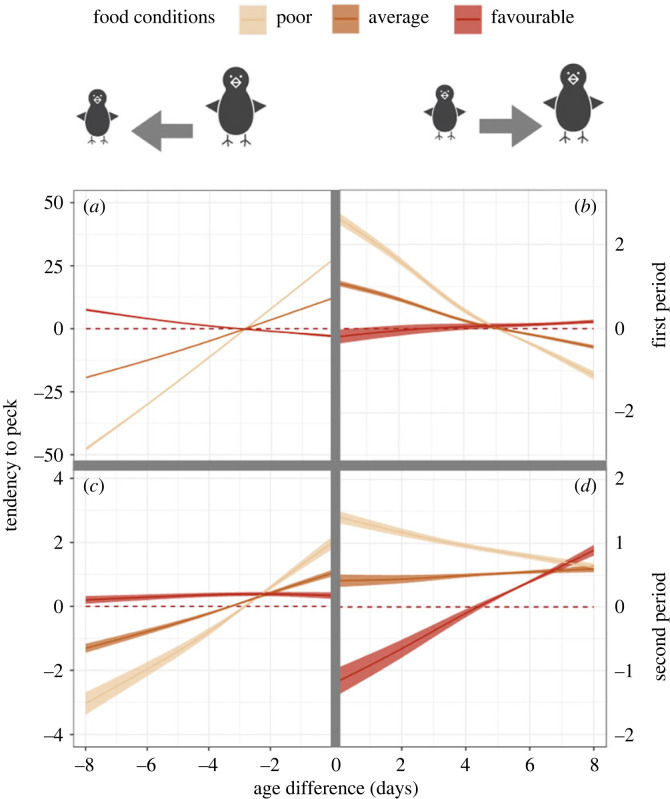
The tendency of red kite nestlings to peck siblings as a function of the age difference (in days) between the aggressor and the recipient for different food conditions (colours) and for the first post-hatching period (*a* and *b*) and the second post-hatching period (*c* and *d*). A positive age difference indicates aggressions targeting older siblings; negative values indicate aggressions targeting younger siblings. The tendency to peck controls for the opportunity to attack, with positive values indicating that the target was preferentially attacked, negative values indicate avoidance of the target. The zero line of tendency to peck indicates pecking behaviour as expected by chance. Note that comparisons between panels is qualitative (avoidance or preference compared to a random pattern), rather than quantitative, as the absolute values of tendency to peck depends on the absolute number of observed pecks. An age difference of 0 corresponds to different predictions at the left and right panels depending on the identity of the attacker and recipient (S, M or J). Solid lines and shaded areas represent the predicted mean value and the bootstrapped 95% confidence interval (*N* = 100 permutations), respectively.

In the first period, nestlings attacked each other with a mean frequency of 203 times per day (s.e. = 44.7). This frequency was lowest under average food conditions (*N* = 115 pecks/day, s.e. = 27), increased under poor food conditions (*N* = 141 pecks/day, s.e. = 42.2) and was highest in days of favourable food conditions (*N* = 186 pecks/day, s.e. = 140). The most frequent attacks (69% of the total number of pecks, *N* = 144 pecks/day, s.e. = 39.6) were directed from the senior to the middle nestling, while 14.4% of the pecks came from the senior towards the junior (*N* = 25.9 pecks/day, s.e. = 5.5). A total of 11.2% of the attacks were from the middle to the senior (*N* = 22.5 pecks/day, s.e. = 6.1) and 4.7% from the middle to the junior (*N* = 9.54 pecks/day, s.e. = 2.9). The junior nestling attacked 0.6% and 0.04% of the times its senior (*N* = 1.3 pecks/day, s.e. = 0.4) and middle (*N* = 0.1 pecks/day, s.e. = 0.09) siblings, respectively (figure 1, panel above). Senior nestlings were more likely to carry out attacks when food conditions were favourable (S to M: *N* = 156 pecks/day, s.e. = 131.1; S to J: *N* = 21 pecks/day, s.e. = 13.7), whereas M tended to attack more frequently in situations of poor food availability (M to S: *N* = 20.5 pecks/day, s.e. = 8.8; M to J: *N* = 7.4 pecks / day, s.e. = 3.8). Under poor food conditions, aggression was strongest among closely hatched siblings, as suggested by the close competitor aggression pattern observed, with senior siblings attacking siblings of similar age substantially more than expected at random, while junior siblings were strongly avoided as the age difference increased (figure 2*a*). Under favourable food conditions, senior siblings were not interacting significantly differently from random along the hatching age gradient, resembling a downward heuristic aggression pattern. The attacks from junior siblings (which were rather infrequent) were in line with those of their older peers, where under favourable food conditions the pattern did not differ from random. However, under poor food conditions, junior siblings disproportionally targeted close-aged peers and avoided their much older ones (figure 2*b*).

In the second period, the daily mean of pecks strongly decreased by 67.6% to 65.9 (s.e. = 22). This changed according to food availability as in the first phase, with lowest mean daily pecks recorded under average conditions (*N* = 31.8 pecks/day, s.e. = 11.9), followed by poor food conditions (*N* = 56 pecks/day, s.e. = 46.7) and favourable food conditions (*N* = 72.1 pecks/day, s.e. = 39.6). S attacked M 42.1% (*N* = 27 pecks/day, s.e. = 12.9) and J 7.0% (4.6 pecks/day, s.e. = 2.1) of the times that aggression was recorded, respectively. M attacked S 25.1% (16.6 pecks/day, s.e. = 10) and J 21.8% (*N* = 14.4 pecks/day, s.e. = 9.8) of the times, while J attacked S 3.8% (*N* = 2.5 pecks/day, s.e. = 0.8) and M 0.2% (*N* = 0.12 pecks/day, s.e. = 0.08) of the times (figure 1, panel below). While in poor food conditions, S remained the main actor of aggression towards M (*N* = 51 pecks/day, s.e. = 48.3), under favourable food conditions M showed increased aggression towards both its siblings (M to S: *N* = 29.5 pecks/day, s.e. = 19.5; M to J: *N* = 27.6 pecks/day, s.e. = 18.8) (figure 1, panel below). Although the absolute dyad interaction frequencies were considerably reduced compared to the first period, the tendency to peck across age differences showed similar patterns, with a close competitor pattern under poor, and a downward heuristic pattern under favourable food conditions (figure 2*c*). Junior siblings targeted rather than avoided their older peers at any age difference, except under good food conditions when they avoided close competitors (figure 2*d*).

### 3.2. Food allocation

**Figure 3.**
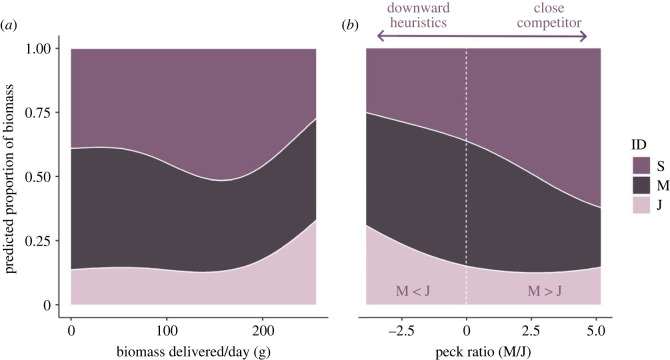
Predicted proportion of food biomass allocated to the senior (S), middle (M) and junior (J) siblings in relation to the daily total biomass delivered to the nest (*a*), and in relation to the prevalent aggression pattern (*b*). In (*b*), the prevalent aggression pattern is denoted by the log-ratio between the attacks received by nestling M and nestling J, respectively. A positive ratio indicates close competitor competition, with fight mostly occurring among close competitors (S and M), while a negative one indicates downward heuristics patterns (i.e. attacks homogeneously distributed among nestlings). Values predicted for the mean age of the oldest nestlings (12.4 days) are shown.

On average, parents delivered daily 73.2 g (s.e. = 8.87 g) and 120 g (s.e. = 17.1) of biomass in the first and second period, respectively. Food allocation among nestlings changed as more biomass was delivered to the nest and depending on which was the dominant aggression pattern (electronic supplementary material, tables S1 and S2; figure 3). When biomass delivered to the nest was low, chick S received ca. 40% of the share, chick M ca. 47% and chick J only 14%. This changed as food biomass delivered to the nest increased, with the senior nestling receiving the largest portion of the allocated food in average conditions (S ca. 50%, M ca. 37% and J ca. 13%; M estimate = −1.31, 95% Credible Intervals (CrI) −1.71 to −0.92; J estimate = −1.97, 95% CrI = −2.49 to −1.49) and the junior then steeply increasing its share when biomass delivered to the nest was high (34% of the total biomass; J estimate = 0.97, 95% CrI = 0.62 to 1.36). This shift in food allocation with biomass delivered was mirrored by an analogous shift in aggression patterns, with nestlings receiving almost equal proportions of food when downward heuristics patterns were expressed and S being allocated most food as close competitor patterns became more common (M estimate = −0.32, 95% CrI = −0.51 to −0.12; J estimate = −0.31, 95% CrI = −0.59 to 0.08).

## 4. Discussion

Our study provides a novel dynamic view of the mechanisms that govern dyadic within-brood sibling aggression patterns. We expanded a recently proposed null-model-based approach to model aggressive dyadic interactions in other contexts [23,24,27], by including differences in hatching rank and variation in food provisioning. This allowed us to test whether changes in food conditions shape the emergence and the dynamics of aggression patterns. Because nestling aggression patterns evolved to establish dominance over resources [46], we examined how the same variation in food provisioning translates into food allocation patterns.

We show that in asynchronous broods, close competitor and downward heuristic aggression patterns alternated in relation to food conditions in the nest and changed from first to second period. In the first period, close competitor strategies are pervasively adopted by both senior and junior individuals under poor food conditions. Conversely, downward heuristic patterns arose when food was abundant, indicating an early stabilization of size-based hierarchies under poor food conditions. In the second period, when the number of aggression events decreased, seniors remained consistent in their aggression patterns, attacking their close competitors under poor food conditions but switched to downward heuristic under favourable food conditions. By contrast, juniors directed their aggressions towards older siblings suggesting that aggression patterns help maintain the dominance roles while food allocation is adjusted to the current amount of available food. These dynamics in aggression patterns promoted a hierarchical food allocation under poor food conditions and an equal food allocation under favourable food conditions. Our results suggest that the dynamic adjustments of aggression patterns to daily food conditions represent the underlying mechanism to regulate facultative brood reduction and to optimize developmental rates, likely maximizing the nestlings' inclusive fitness across nestling phases in variable environments.

Theory predicts that an early onset of dominance hierarchy in social groups helps to save energy in contests later on [47,48]. Indeed, red kite senior siblings vigorously engage in aggressive interactions when they're young, but in the second period, the frequency of aggressive interactions is considerably reduced. The early aggressive behaviour against their close competitor is suggested to be a strategy to stabilize the hierarchy [49]. This pattern is even more evident under poor food conditions, which is in line with the idea that hunger (subjective resource value), together with the probability of winning a contest (resource-holding potential) can affect aggression patterns [50]. The scarcity of resources may, in fact, boost the motivation of senior nestlings to engage in fights because obtaining dominance over food when resources are limited entails greater advantages than under favourable conditions [51,52]. By being strongly avoided by much older siblings, and in return by avoiding them, junior siblings can prevent wasting energy and increase their survival chances in case food conditions improve in the near future. If food conditions remain poor, junior siblings will be selectively starved and die without the need for senior siblings to invest energy into competitive fights [53]. After surviving the first period, the survival probability of junior siblings increases, representing an additional benefit for their older siblings by increasing their inclusive fitness [28,54]. As the nestling phase progresses, the persistence of the close-competitor pattern in poor, and downward heuristic pattern in favourable food conditions suggests that the investment in competitive interactions with close-aged peers might be important to maintain the dominance structure [47,55].

Under poor food conditions—when close competitor strategies were favoured—nestling J barely received any food. Yet, as food conditions improved and downward heuristic patterns arose, the proportion of food consumed by the junior nestling increased. This relation between aggression patterns and food allocation suggests that as food conditions improve, marginal nestlings gain more food but are more likely the target of aggression from their siblings. We see three potential mechanisms explaining this. First, parents, who are able to judge the ongoing food conditions actively allocate this ‘extra’ food to the marginal nestling, as previously reported in black kites [56], exposing it to increased aggression by being in the crosshair of food distribution. Second, marginal nestlings increase their effort to receive food (by increased begging early on and scramble competition later) and become more exposed to aggression as the pay-off for their efforts exceeds the costs [57], suggesting an ability of nestlings to adjust their behaviour to their current environment (‘Bayesian update’, [58]). Last, regardless of how more food is allocated to the marginal nestling, the downward heuristic pattern purely reflects a way to maintain a size-based dominance hierarchy when enough resources are available [59,60]. While nestlings start transitioning from being solely dependent on parental feeding to independently consuming prey brought to the nest [36], aggression patterns seem to persist from the first to the second post-hatching period. This suggests that the shift towards a downward heuristic aggression pattern in favourable food conditions is unlikely to be influenced by parental allocation preferences. Rather, the change in dyadic aggression patterns is more probably the result of shifts in the cost-benefit ratio of aggressive behaviour, independent of parental allocation strategies. Although the precise mechanism responsible for this change remains unclear, it is evident that marginal nestlings must bear the cost of aggression to benefit from the energy surplus during the downward heuristic aggression phase. Our results therefore highlight that the occurrence of different aggression patterns during nestling development of red kites allow for survival, for achieving normal growth rate or even for catch-up growth of junior nestlings (for catch-up growth, see [61,62]) after periods of poor food conditions.

The aggression patterns we found might have specific consequences for the siblings beyond the direct consequences operating during the nestling period. These consequences can occur according to the obtained dominance rank [63–65] but see [66], but can also vary among individuals of the same rank. First, senior nestlings incur high costs through the repeated occurrences of close-competitor aggression especially under poor food conditions. These costs associated with the establishment and maintenance of dominance can have long-lasting consequences [67]. Second, nestlings with large age differences were avoided and refrained from engaging in fights with their older siblings, while they became more involved both as targets and as attackers as conditions improved. This means that for marginal nestlings if they survive, food limitation imposes short- and long-lasting costs by both reducing growth rates and reducing the development of competitive abilities. On the one hand, in fact, reduced growth rates due to food limitations as well as catch-up growth are associated with long-lasting consequences such as increased metabolic rate as adults [68] and telomere shortening [69,70]. On the other hand, individuals who experience reduced social skill development may exhibit poor performance in contest resolution contexts, which can have significant implications for their overall breeding success and survival [71,72]. Hence, we suggest that sustained scarcity of food during the nestling period entails aggression-mediated costs for all nestlings in a brood, but that these costs differ between nestlings hatching ranks. In general, the dynamic aggression patterns in red kites ultimately modulate the overall effect of environmental conditions on quality differences within the brood over three axes. First, they regulate food allocation within the brood in environments with high temporal variability in the flow of food to the nest resulting in intra-brood variation in body condition at fledging and the associated carry-over effects. Second, they mediate the achievement of a hierarchical position within a nest, which, when coupled with adverse food conditions, can impact the development of social abilities and the establishment of dominant and subordinate personalities. Lastly, they regulate the way a hierarchical position is achieved by increasing costs for the close competitors, especially when food conditions are poor, which can have significant fitness consequences.

Adopting a dynamic network approach in the study of interactions in social systems provides new research avenues across different scales of model systems [73], and one of the most exciting may be represented by parent-offspring conflicts in family groups. Variation in sibling aggression has been traditionally viewed as originating from species-specific patterns of hatching asynchrony creating predictable aggression patterns under changing food conditions (reviewed in [16]). Here we addressed the claim to rethink developmental behavioural strategies as dynamic processes [74] by using a dynamic network approach. We relaxed the boundaries of traditional frameworks in light of recent studies showing the existence of variable aggression patterns within and between social groups of the same species [24,27]. Our results suggest that sibling aggression in red kites aims at creating a size-based dominance hierarchy, but the costs associated with this process as well as food distribution are driven by transient food conditions. Future studies should aim at extending sibling interaction networks and food distribution models to parental behaviour, from which we could uncover parent–offspring behavioural processes beyond that of competition which are crucial for the understanding of the underpinning mechanisms and role of family life [75]. The integration of family systems will further allow us to examine how extrinsic factors such as environmental conditions during the rearing period affect the entire family dynamics and how this translates into fitness consequences.

## Data Availability

Data and code are publicly available at: https://doi.org/10.5281/zenodo.7913085 [76]. Electronic supplementary material is available on Figshare [77].
